# A guide for evaluation of online learning in medical education: a qualitative reflective analysis

**DOI:** 10.1186/s12909-021-02752-2

**Published:** 2021-06-10

**Authors:** Nourhan F. Wasfy, Enjy Abouzeid, Asmaa Abdel Nasser, Samar A. Ahmed, Ilham Youssry, Nagwa N. Hegazy, Mohamed Hany K. Shehata, Doaa Kamal, Hani Atwa

**Affiliations:** 1 Medical Education Department, Faculty of Medicine, Suez Canal University, Ismailia, Egypt; 2 Ibn Sina National College, Jeddah, Kingdom of Saudi Arabia; 3 Ain shams University Middle East North Africa FAIMER Regional Institute, Ain shams University, Cairo, Egypt; 4 Pediatrics & Head of the Pediatric Hematology Unit, Cairo University, Cairo, Egypt; 5 Family Medicine Department, Faculty of Medicine, Medical Education and human resources development Center, Menoufia University, Shebin Elkom, Egypt; 6 Faculty of Medicine, Helwan University, Helwan, Egypt; 7 College of Medicine and Medical Sciences, Arabian Gulf University, Manama, Kingdom of Bahrain

**Keywords:** Online learning, Standards, Qualitative

## Abstract

**Background:**

With the strike of Covid-19, an unprecedented rapid shift to remote learning happened worldwide with a paradigm shift to online learning from an institutional adjuvant luxury package and learner choice into a forced solo choice. This raises the question of quality assurance. While some groups have already established standards for online courses, teaching and programs yet very little information is included on methodology of their development and very little emphasis is placed on the online learning experience. Nevertheless, no work has been done specifically for medical education institutions.

**Aim:**

To develop a set of descriptors for best practice in online learning in medical education utilizing existing expertise and needs.

**Methods:**

This work utilizes a qualitative multistage approach to identify the descriptors of best practice in online learning starting with a question guided focus group, thematic analysis, Delphi technique and an expert consensus session done simultaneously for triangulation. This was done involving 32 institution in 19 countries.

**Results:**

This materialized into the development of a set of standards, indicators, and development of a checklist for each standard area. The standard areas identified were organizational capacity, educational effectiveness, and human resources each of which listed a number of standards. Expert consensus sessions identified the need for qualification of data and thus the development of indicators for best practice.

**Conclusion:**

Standards are needed for online learning experience and their development and redesign is situational and needs to be enhanced methodologically in axes that are pertaining to the needs of the education community. Taking such axes into consideration by educators and institutions will lead to planning and implementing successful online learning activities, while taking them into consideration by the evaluators will help them conduct comprehensive audits and provide stakeholders with highly informative evaluation reports.

**Supplementary Information:**

The online version contains supplementary material available at 10.1186/s12909-021-02752-2.

## Background

There is an increasing interest in the use of online learning in medical education from early undergraduate years through residency and fellowship training, and in continuing medical education (CME) [1].

This “disruptive innovation” of educational format is constantly evolving with different attempts and experiences to design, implement, assess, monitor, and evaluate [1]. With the strike of Covid-19, an unprecedented rapid change to remote learning happened worldwide with a paradigm shift to online learning from an educational option to the only existing solution [2, 3].

This raises the issue of quality assurance since the conversation is turning from remote instruction as an emergency fix to a new ongoing reality in education.

Recently, there have been efforts to standardize the necessary criteria for developing new content, adopting efficient teaching methods for online learning and establishing resources, yet no specific attempts have been done within the context of medical education [4]. Among of the professional groups that have been working to reach this goal are the International Organization for Standardization (ISO) [5] and Association of Medical Education in Europe (AMEE) [6].

Bari and Djouab [7] found that the existing standards or frameworks need to be modified to fit with the context of specific institutions. Despite all the existing attempts, there remains a need to generate operational guidelines to guide execution of online education focusing on building and evaluating the experience in medical education settings.

Many of the existing literature on online standards develops standards that address quality of content [5] with very little focus on policies and processes.

Thus, the purpose of this work is to address the gap in the quality assurance guidelines of online learning. These standards for online learning experiences, as a comprehensive set of criteria are important to establish confidence in online learning among stakeholders and to facilitate structured and objective comparisons between various offered courses [8]. Such standards and their indicators can also support the design of a guide for peer-reviewing and self-assessment for further improvement of online education.

## Methods

Under the interpretivist paradigm, we used a deductive qualitative grounded theory approach [9] aiming at creating a deeper understanding of the perceptions of medical educators and to explore the essence of their online experiences. This work applies the grounded theory in a longitudinal approach through four phases.

### Phase 1: virtual focus groups

Two virtual focus group discussions were conducted. A convenient non-probability sample of faculty members in the regional medical schools were officially invited to participate. A total of 32 Universities from 19 countries participated. They varied in gender, specialty, academic rank, and affiliation. Precautions were taken to guarantee both the anonymity of the participants and the confidentiality of their contributions to the discussions (e.g., participants’ contributions to the focus groups were anonymized and participants’ identities were hidden during data analysis).

Thirty faculty members attended each focus group discussion. This discussion was split into 5 groups and lasted for 90 min. Each group consisted of 6 faculty members and was moderated by one of the authors. The focus group discussions followed a deductive approach where the hypotheses and themes were derived from the data. In the focus groups, the moderators used a developed focus group guide that included questions aiming at exploring participants’ views on what makes a good online learning experience reflecting on the transition phase they already went through post COVID-19.

Questions in the focus group guide covered three major themes: organizational capacity, effective learning and assessment, and online learning.

The kickoff of the focus group was in the form of leading sentences and questions that are summarized in Table 1.

**Table 1:** Sentences and questions used to guide focus group discussions

• *From your previous experience, what is the role of institutional leadership in the success of online learning?*	
• *What kind of resources did you feel were necessary to conduct a successful online learning experience in your school?*	
• *What elements in the organizational bylaws need to be added or adapted to cope with the shift to online learning?*	
• *What are the essentials for shifting face-to-face programs into online format?*	
• *What are the elements required to create a motivating environment in online learning that ensures students’ engagement?*	
• *In your opinion, what are the criteria of choosing effective online assessment?*	
• *What are the most important evaluation criteria specific to online rather than face-to-face learning?*	
• *What are the attributes/capabilities needed by faculty, students, and administration for online learning?*	

### Phase 2: generation of standards and indicators

The focus group data was analyzed thematically and interpreted to identify descriptors of best practices in online learning. This was done by the authors. The focus group data was analyzed thematically, and saturation was confirmed by the authors in a series of 3 virtual meetings each lasting for 3 h. The themes were interpreted to identify descriptors of best practices in online learning.

### Phase 3: Delphi technique

To get the consensus of the experts on developed indicators and descriptors of best practice, a survey was developed based on the focus group discussion findings and was pilot tested on a group of 5 respondents.

The survey was distributed using the Delphi approach. A two round online Delphi survey was conducted from October 2020 to January 2020.The Delphi panel consisted of 15 regional medical educators purposely selected based on their experience in online teaching and in managing quality standards and who had not attended any of the focus group meetings. Over subsequent rounds of the Delphi, participants were invited to approve the quality area with its suitable indicators. This technique was repeated until 92% of the standards were approved by 100% of the panel.

### Phase 4: expert opinion consensus session

A purposive sample of national and international experts in online learning were invited to participate in an expert opinion consensus session. An ‘expert’ was defined as a person having different experiences and expertise in online learning from a range of different contexts. Experts were short-listed by members of the research team and were invited to contribute their time. The experts’ opinions regarding the descriptors were aggregated and summarized. Forty-one experts were formally invited to attend a 1 day expert virtual panel meeting. The goal was to reduce the range of responses and arrive at something closer to expert consensus. Before their attendance, experts were given clear, written guidance on the objectives of the meeting and the required output of the expert panel – to review the standards of quality in online learning and its related indicators.

The credibility of this study was established through “analyst triangulation” to confirm and verify the conclusions drawn from the analysis. This required involvement of external reviewers (Experienced medical educators with varied backgrounds) who worked together to analyze the transcripts until consensus was reached. This analysis process helps to facilitate discussion and clarify possible blind spots. So phase 3 and phase 4 were conducted in parallel to achieve this triangulation.

#### Data collection and analysis

All focus groups were audio recorded and transcribed anonymously by the authors. The transcripts were checked for accuracy and unclear data identified was excluded. The moderator field notes and reflections on the transcribed data were attached to provide context. Two of the authors; NW and EA independently reviewed and analyzed the focus groups transcripts using a deductive approach. Thematic analysis was applied to identify common themes. Delphi technique analysis was done by calculating the percentage of consensus of each standard and repeated until there was no significant difference between percentages calculated.

Ethical approval was obtained from the Research Ethics Committee (REC) of the Faculty of Medicine, Ain Shams University.

## Results

The results of this qualitative study will be presented into three main sections as following:

### Phase 1: thematic analysis of the focus group contributions

**A number of themes emerged from analyzing participant contributions in the focus group as follows:**

#### The role and attributes of the leaders for successful online learning

Many participants suggested that leaders should set rules and be decisive especially in time of crisis. Encouraging teamwork and including everyone are the cornerstones for achieving goals. Another important attribute is flexibility and how to cope with different personalities and work requirements. Visionary leaders who predict the future and act proactively to suggest solutions, crisis management, support and sustainability were added as important roles of the leaders. One of the participants added that ‘*effective leadership requires receiving feedback from significant stakeholders including the students and they must be seen and heard for highly relevant feedback’*. Proper communication can help in overcoming several obstacles as one participant mentioned ‘*Leaders should provide early and continuous student and faculty orientation about milestone in the learning experience.’*

#### Resources needed to conduct a successful online learning

In their discussions, participants emphasized the importance of resource allocation for online learning. These resources include user friendly learning management systems (LMS), internet services, ready-made or self-generated digital tools and equipment to support the online learning. Moreover, an important resource are the personnel involved in the online learning. They also added the importance of having a supportive IT team and conducting a well-organized faculty development program. One participant said, *“My college had a well-established LMS that helped us a lot during the pandemic transition”.* Another one said, *“We used to upload our lectures on Moodle with the help of the IT team. When the pandemic started, this step helped us to overcome the chaos that faced other colleges”.*

#### Institutional bylaws

The participants’ responses varied regarding the modifications of the vision, mission, and bylaws. Some of the participants highlighted the importance of revising the institutional mission and vision. One participant noted that *“We need to revise the mission and vision to cope with the changes that we are facing in the post-Covid era that may be reflected on the graduates’ competencies*”. Others confirmed that the mission and vision will not be changed for the use of a new mode of learning. One participant remarked that *“Our mission and vision can be achieved despite the change in the educational strategy. Therefore, introducing blended learning or online learning as a mode of learning may affect the teaching strategies but not the mission and vision”*. Another participant confirmed *“The mission and vision should be more generic, but I think that some points should be added to the bylaws, as including the ratio of online to face-to-face learning, the assessment plan, and attendance ratio, in addition to a clear description of online learning competencies and required staff and faculty qualifications”*.

#### Key points to consider while shifting face to face programs into online format

Participants stressed that institutions must start with development of the skills and knowledge of their faculty. Faculty should understand the difference between face to face and online learning. The role of faculty, the nature of the content and instructional methods will change with this shift. Also, faculty and students needed to develop some essential skills to cope with this transformation. A participant mentioned *“I was assigned to moderate sessions on different platforms as Zoom and Telegram and upload recorded lectures on Moodle. The training provided by my institution before and during this transition was my only guidance to perform this role effectively”.* Materials should be simplified, interactive and motivating. Proper platforms or LMSs are important to conduct successful online learning since it is the ‘vehicle for all the activities’. Use of all the available tools to engage students and create interactive activities such as whiteboard, share-screen, assignments, e-portfolio, online quizzes, online discussion forums. Still there are difficulties with conducting practical and clinical sessions. Virtual reality and simulation may help in this point, but funding will remain the main obstacle.

Guidance is required in a comprehensive way in online learning to engage the students and avoid isolation. It is important to provide the students with different alternatives that facilitate and ensure their engagement and participation even with poor internet connections particularly in rural areas. In online learning, mentorship and coaching are needed even more than in face-to-face learning. Finally, finding alternatives for clinical and practical skills teaching is a big challenge. Formative assessment and feedback are also critical points to consider.

#### Creating a motivating/engaging environment in online learning

Student engagement was a major problem that faced most of the universities last year as mentioned by many participants. Therefore, participants highlighted some important practices that should be considered while implementing online learning. One participant added the need for the use of more formative assessment to keep the students engaged: *“Students become more engaged when they are about to have exams…”.* However, there is a need for redesigning and adapting teaching and learning materials to fit the new learning environment as one participant reported that *“We need to redesign our lectures to adapt to the new era of online learning…”.*

In their discussions, participants linked the interesting content with student engagement. Therefore, they recommended the use of gamifications, quizzes, and the use of Multimedia learning principles *“If we followed multimedia learning principles, that would help in both instruction & assessment…”*. Though it is still of high importance to select the suitable platform.

According to the participants, selecting the best model for learning may help the students’ development of clinical reasoning skills with the help of scenarios, interactive diagnostic reasoning softwares and virtual simulation.

Student centered approaches and methods can be of great benefit especially in online learning. According to one of the participants *“It will allow the students to lead and this may help them to feel secure”.* Another participant added *“Engaging the students with a student-centered activity will get them out of isolation and will help the faculty to detect any student that was left behind”.* Additionally, the use of group work learning/teaching methods as online TBL may foster the development of a collaborative environment.

#### Criteria of effective online assessment

Assessment becomes one of the most important dilemmas when shifting to online learning. Ensuring validity and reliability of the exam is a challenge when examinations are done at a distance. *“Student Assessment in online learning should be innovative, secure, out of the box, creative and aligned with the teaching methodology.”*

There is also the concept of accessibility and how the exam is made available to students. This requires availability of alternatives and ensuring flexibility of format. “*Assessment methods used in online learning should be open-book exam, case-based scenarios, single best answer, assignments, virtual OSCE, pattern recognition sessions e.g., histopathological slides, X-rays identification, clinical signs. Choosing the suitable online assessment method depends on the nature of the course, the available resources, student number and student staff ratio”.*

#### Criteria of online learning evaluation

Types of program evaluation that seemed popular among participants were process and outcome evaluation. However, participants emphasized the need for a comprehensive model of evaluation as the CIPP model [10] because of the complexity of the online learning. *“Merging more than one model of evaluation is indicated and highly important in online learning evaluation”* was added by one of the participants.

When the participants were asked about the differences between face-to-face course evaluation and online course evaluation, one of the participants mentioned “*Modifying and updating the traditional course evaluation surveys to include evaluation of learning management systems (LMS), connectivity and technical support”.*

*“Collecting the contact details of the registered students is an important step to facilitate online courses evaluation”* was added by one of the participants.

Using different data collection tools in online formats was recommended by participants, surveys and students quizzes are preferred.

Participants suggested that student engagement in online learning should be evaluated in terms of student interaction, performance, and assessment. *“Learning management systems (LMS) analytics such as submission of assignments, synchronous sessions attendance and dropout rate are indicators for students engagement”.* Finally, finding the suitable benchmark program and logic model for evaluation is of high importance as well as the need for external peer review to validate the evaluation process.

#### Important evaluation questions

Participants suggested some areas and questions that should be covered in evaluation. Examples of suggestions and quotes are:

- Evidence to prove learning: *“Did the assessment match the curriculum?”, “Does the program/course help students to learn and grow?”*
- Student and staff satisfaction: *“Are the staff satisfied with the online learning experience? Do they prefer face to face learning?”, “Did institution meet with individual variation?”*
- Management system analytics and faculty performance: *“Does online learning help tutors/faculty to be better teachers?”*
- Student interaction, and student performance including the analysis of quiz grades, dropout rate, delay in assignment submission, discussion participation: “*Does the program foster student interaction?”, “If this course/program is optional, would students apply for it?”, “What is the success rate of the students?”, “What is the dropout rate of the students?”*

#### Faculty, students, administration attributes in online learning

Most of the participants discussed the competencies faculty members should have in online learning. They noted that all faculty members should be skilled in using technology and online platforms and show creativity and innovation. Faculty also should show proficiency in online communication, course design, online assessment, time management and in engaging students in an online learning environment. One participant added *“I and my students are suffering the online learning isolation. So, I think using student-centered approaches can be our savior in this situation”*.

Beside the proper use of technology in learning, engagement, critical thinking, collaboration, teamwork and communication, students should also know how to manage learning in an online context. One participant added, *“We have to equip our students with other skills than medical ones, as self-regulated learning, time management, setting goals, and how and when to seek help”*.

The role of administration in assuring quality of online learning is an integral one. Participants nominated different attributes that will help administration maintain quality online learning including management skills, technical skills, strategic planning attributes and risk management, decision making, ethics and professionalism, communication, monitoring and evaluation.

### Phase 2: formulation of descriptors of best practice

The focus group contributions were analyzed and reformulated by the authors into quality standards and indicators. Three main quality areas were identified: Organizational capacity, Learning and assessment and Human resources. The standards were designed as follows:

### Organizational capacity

#### Governance

*School Leadership is accountable and committed to support and lead the institution to delivering quality online education.*

##### Indicators

1. Leadership encourages a collaborative environment to plan, implement and monitor the quality of online learning activities.
2. Leadership shares and cements the values, beliefs, and the operational expectations for a quality online learning.
3. Leadership holds themselves accountable to disclose accurate information about the recruitment process, policy, fees, courses/programs, and reports.
4. Leadership demonstrates proactive understanding and analyzing organizational needs to deliver effective online education.
5. Leadership creates a culture of acceptance and encouragement for online learning.
6. Leadership delegates responsibility to multidisciplinary teams and facilitates their work to implement and monitor online learning activities.

#### Resources

*Resources for the online learning are allocated in a fair, reasonable manner that responds to the identified needs.*

##### Indicators

1. Presence of a learning management system (LMS) that ensures user-friendly and secure online environment.
2. Presence of accessible Internet services
3. Presence of digital tools that are aligned with the educational needs of learners.
4. Presence of the equipment that support successful online learning.
5. Presence of trained technical support team.
6. Financial resources allocated to online learning.
7. Provisional needs documents are available designed annually and approved by proper authorities.
8. Budget is well-managed in a transparent and documented way.

#### Organizational bylaws (regulations)

*Bylaws clearly define the administrative issues, credit points calculation and the roles and responsibilities of team members.*

##### Indicators

1. Presence of written policies & procedures or all online courses
2. There is a defined and documented process related to the online programs
3. There is a documented clear policy governing the ongoing training and support to the working staff.
4. All students have equitable access to the online learning resources.

### Effective learning and assessment

#### Program

*The program has a clear robust design that respects the school vision, mission, and values and that demonstrates a clear understanding of the nature of the required graduate attributes.*

##### Indicators

1. There is an approved, updated and well-constructed longitudinal online education plan that includes sufficient data to support decisions and is aligned with the educational program.
2. There are aligned and cascaded goals: strategic, long term, intermediate and short-term goals.
3. There is clear identification of the required resources to ensure sustainability of the online programs and courses.

#### Course design

Courses have a clear robust design that respects the school vision, mission and values with a clear distinction of the allocation of online teaching/ learning practices.

##### Indicators

1. Courses have clearly stated learning objectives/ competencies that are aligned with the organization goals
2. The course learning objectives or competencies describe outcomes that are measurable.
3. The selected contents are UpToDate, related to learning goals and follow the legal requirements (ownership, intellectual property, copyrights)
4. The instructional materials contribute to the achievement of the stated learning objectives or competencies.
5. Online instructional methods and tools support active learning, student involvement, support interaction amongst students and between instructors and students and are based on recent best practices.
6. Online instructional methods are variable and support development of higher order thinking.
7. The relationship between learning objectives or competencies and course activities is clearly stated.
8. There is use of digital tools that best support students’ involvement and better understanding of the learning material.
9. Learning and assessment schedules are clear, applicable, and fair for all students.
10. Online student Assessment methods planned are clear, fair for all students and include frequent formative assessment with feedback and summative assessment with clear and transparent reporting.

#### Course delivery

*Courses should be delivered in the safest most accessible way providing standardized learning opportunities.*

##### Indicators

1. There is a plan for frequent evaluation that is approved and implemented with identified data collection methods (e.g., observation, questionnaires, focus group).
2. There is a well-organized plan for delivery with a backup.
3. A troubleshooting and complaint policy and procedure exists and is announced and used by learners.
4. Designed learning activities are implemented with minimal deviation from plans.
5. Technologies required in the course are readily obtainable.

#### Student assessment

*Student assessment to measure student achievement using multiple assessment methods that align with the learning objectives and the instructional methods. Data from assessment is evaluated and feeds into educational decision making.*

##### Indicators

1. Digital tools are used to ensure secure, fair, valid, and applicable assessment.
2. There is use of multiple assessment methods to measure cognition, skills, and attitude of the students.
3. There is use of frequent formative assessment with feedback for better learning.
4. There are clear reports after the summative assessment.
5. There is a plan for academic counseling that is clear, manageable and is executed.

#### Evaluation

*Educational monitoring and evaluation plans are available with clearly assigned evaluation questions, key performance indicators and assigned personnel. The plan is implemented and the information it generates feeds into the educational replanning.*

##### Indicators

1. There is documented continuous monitoring and evaluation for the online learning materials/ process by internal reviewers to collect and analyze data for continuous improvement. (about LMS, Faculty performance and satisfaction, and Students’ Engagement, Satisfaction, and Achievement)
2. There is documented periodic evaluation by external reviewers to validate the internal evaluation process and assess the goal achievement.
3. There is disclosure of the evaluation results with the stakeholders.
4. Data is used to drive decisions for continuous improvement.

### Human resources

*The organization has personnel who can manage the educational process effectively and who are under continuous monitoring and development.*

#### Indicators

##### Faculty

1. There is a wide variety of professional development activities for the faculty pertaining to skills needed for online education.
2. There is timely and effective technical support to the faculty.
3. There is timely, frequent, and constructive feedback about instructor performance.
4. Faculty have an opportunity to add to their professional portfolio within online learning in the school.
5. The number of assigned faculty is reasonable, sufficient, and aligned with the student number and educational activities.
6. There is a clear definition of faculty roles and responsibilities.

##### Students

1. There is briefing and orienting the students about the accessibility and availability of the online learning resources and digital tools.
2. Equity and accessibility to technology to all students is ensured.
3. There is timely and effective technical support to students to overcome limitations of technology & computer literacy.
4. There are guidelines for student-teacher and student-student communication.

##### Administration

1. There exists a supporting administration team that is reasonable and aligned with the educational processes, number of students, number of faculty etc.
2. There is a solid development plan for administration of the online learning program.
3. There is a clear role definition for administration.
4. There is a definite pathway for troubleshooting and for complaints for administrators in the program.

### Phases 3 and 4: expert consensus session response and Delphi technique

The abovementioned descriptors of online learning were handled by experts, and the following results were achieved:

A- All suggested standard areas were agreed upon with no further additions or amendments by 100% of experts, except the following standards, which were accordingly amended:

## Digital tools are used to ensure secure, fair, valid, and applicable assessment

### Suggested amendment

Digital tools need to be more specified into KPIs (e.g., number of trained faculty on the digital tools of assessment, blueprinting to ensure content validity).

## There is a plan for academic counseling that is clear, manageable, and is executed

### Suggestion amendment

There is a plan for academic counseling that is clear, manageable, and supported by the administration.

B- A set of other standards were proposed to be added (Table 2).

**Table 2:** Suggested amendments to the standards done in the consensus session

Quality areas	Suggested indicators
**Organizational bylaws**	1. Course specifications are to be linked to the methodology of the online teaching and the method of assessment. 2. Define the platforms used for online learning. 3. Presence of a clear policy for training the students and the staff. 4. A guide for the proposed online methodologies is to be provided by the SCU.
**Course delivery**	1. Establish an e-library to act as an educational backup. 2. Emphasis formulation of e-learning committee and direct their effort towards the students and students.
**Student assessment**	1. To specify the number and type of assessment tool for each domain separately (e.g., MCQs and essays for cognition, OSPE and DOPS for practical skills). 2. To specify the minimum number or be continuous formative for all types of domains. 3. Specify Corrective actions based on the students results of the formative assessment. 4. Corrective actions based on the students results of the summative assessment to be implemented in the next cycle.
**Administration**	1. There is an established program for continuous faculty training on skills needed for online teaching. 2. There is documented monitoring and reporting on the activities (sessions and exam) 3. There is an established program for student training. 4. There is a clear pathway for feedback analysis for improvement points.

Based on the above findings and recommendations, a set of checklists were developed (Tables 3, 4 and 5).

**Table 3:** Checklist for quality practices in organizational capacity

Checklist for Organizational Capacity	Evidence Collection Method
**Governance**	
*Evaluation questions answered in advance:*	
1. Is there a designated team for OL implementation? 2. Is there a designated team for OL evaluation? 3. Is this team inclusive? 4. Is there a communication plan for online learning? 5. Is there a stated core value for OL? 6. Is this value reflected in daily decisions in OL? 7. Is the Standard operating procedures (SOP) document available and used by faculty? 8. Is there a policy for transparency? 9. Are the issues concerned with recruitments assignments and financial decisions discussed in the faculty meeting? 10. Are decisions regarding acceptance, fees, student selection made in an inclusive manner? 11. Are stakeholder meetings held to test community needs? 12. Are surveys used to collect community needs? 13. Are identified needs translated into learning opportunities? 14. Are online classes treated financially and in workload calculations like face-to-face classes?	1–14: document analysis
*Evaluation questions answered during audit visits*	
15. Is there a standard operating procedure for OL execution? 16. What percentage of the curriculum is taught online? 17. Are some of the faculty meetings conducted online? 18. Are there privileges given to online tutors?	15–17: observations 18: interviews
**Resources**	
*Evaluation questions answered in advance*	
1. Are the educational tools matched to the objectives? 2. Are there on-campus computers? 3. How many computers are available per student (1 computer for every 4 students)	1: expert document review 2 and 3: observations / documents review
*Evaluation questions answered during audit visits:*	
4. Is there a Learning Management System (LMS)? 5. Is the LMS user friendly? 6. Is this LMS accessible to all students? 7. Are at least 50% of its features utilized? 8. What percentage of the campus is connected to the internet? 9. Are there internet-connected study areas/libraries? 10. Is the data upload speed sufficient to run virtual meetings? 11. Is there a variation in the tools used for education? 12. What additional equipment exists to help OL? 13. Is there a sufficient number of trained technical support teams? 14. Are there financial allocations for OL course design and implementation? 15. Are provisional needs documents available, designed annually, and approved by proper authorities? 16. Is the budget handled by a special unit and is reported to the designated council around a known schedule?	4, 5, 8, 9, 11–13: observations 6 and 7: interviews / focus groups 10: observations and speed tests 14–16: interviews
**Bylaws**	
*Evaluation questions answered in advance:*	
1. Are the institutional mission and vision modified to include online learning activities? 2. Is there a policy and procedure (strategy) document for all online programs and courses? 3. How readily do faculty use this policy? 4. Is there an IPR office? 5. Are there guidelines for copyrights and IPR? 6. Is there a training plan for personnel? 7. Is personnel training related to their promotion? 8. Is there an online library?	1–8: document analysis
*Evaluation questions answered during audit visits:*	
9. Is the online library accessible to students? 10. Are they aware of it? 11. Is this library being used by students and faculty? 12. Is this policy circulated?	9 and 10: interviews 11 and 12: interviews / observations

**Table 4:** Checklist for quality practices in educational effectiveness

Educational Effectiveness Checklist	Level of Achievement (Fully / Partially / Not Achieved)	Evidence Collection Method
**Educational Program**		
*Evaluation questions answered in advance*		
1. Is there an approved, updated and well-constructed online education plan? 2. Is there a matrix mapping of goals of OL against the vision and mission (at least 70% of subsets overlapping)? 3. Is data available to support decisions made in the program? 4. Is the online teaching plan aligned with the program learning outcomes? 5. Are the goals aligned and cascaded: strategic, long term, intermediate and short-term? 6. Is there a clear identification of the required resources for the program?		1–6: document review
**Course Design**		
*Evaluation questions answered in advance:*		
1. Do courses have clearly stated Intended learning outcomes? 2. Is there a mapping matrix for course goals with organizational goals? 3. What is the percentage of online learning activities? (According to the nature of the course and its intended learning outcomes not more than 70% in basic courses and 50% in clinical courses). 4. What is the ratio between face-to-face to online assessment methods? (According to the nature of the course and its intended learning outcomes with consideration to the continuous, formative and summative methods) 5. What is the ratio between synchronous and asynchronous online learning activities? (1:1 is an acceptable ratio based on the requirements of the delivered material) 6. Are the selected contents up-to-date? 7. Are they related to ILOs and follow the legal requirements? (ownership, intellectual property, copyrights) 8. Do online instructional methods support the development of higher thinking skills like analysis, application, synthesis, integration, problem solving, critical thinking, clinical reasoning skills etc.? 9. Is there a faculty guide that shows how tools are applied with a specific targeted objective?		1–9: document review
*Evaluation questions answered during audit visits:*		
10. Are digital tools used to support students’ involvement and overall learning experience? 11. Do Instructional methods encourage student engagement? 12. Are online instructional methods variable to suit all learning styles?		10: focus group 11: interviews / observations 12: observations
**Course Delivery**		
*Evaluation questions answered in advance:*		
1. Are the designed learning activities aligned with course learning outcomes as per the specifications? 2. Was the evaluation plan developed by a team of experts? 3. Was the evaluation plan revised and approved by relevant councils? 4. Are data collection methods identified in the evaluation plan? Are they concise, realistic, and aligned with the evaluation questions? 5. Is there a plan for proper evaluation? 6. Is the deviation from planned schedules kept as limited as possible? 7. Is the synchronous meeting application defined and accessible for students? 8. Is it user friendly? 9. Does the application facilitate learner engagement? 10. Is there an illustrative guide for synchronous meetings published and used by faculty and students?		1–4, 6–9: document review / document analysis 5 and 10: expert review
*Evaluation questions answered during audit visits:*		
11. Are learning activities purposeful and aligned with objectives? 12. Is a technology requirement document disseminated and used by faculty? 13. Are the minimum technology requirements clearly stated and instructions for use provided in a document? 14. Are the schedules present and announced? 15. Is there a complaint box/email?		11–14: focus group / interviews 15: observations
**Student Assessment**		
*Evaluation questions answered in advance:*		
1. Are the used online student assessment methods clear, fair and acceptable for all students? 2. Is formative assessment used at proper intervals (at least one per module)? 3. Do students receive structured feedback after each formative assessment? 4. Is summative assessment planned with clear and transparent reporting? 5. What is the ratio between the continuous and summative assessment with consideration to use different online assessment methods? 6. Does the assessment measure the stated learning objectives/ competences?		1–6: document analysis / interviews
*Evaluation questions answered during audit visits:*		
7. Which digital tools are used? 8. Why were they chosen? 9. Are multiple assessment methods used to measure cognition, skills, and attitude of the students? 10. Are the assessment schedules clear, applicable and fair for all students? 11. Are the assessment schedules announced on the website for all students?		7, 10, 11: observations 8 and 9: interviews
**Evaluation**		
*Evaluation questions answered in advance*		
1. Is there a documented continuous monitoring and evaluation for the online learning materials/ process by internal reviewers to collect and analyze data for continuous improvement? (about LMS, Faculty performance and satisfaction, and Students’ Engagement, Satisfaction, and Achievement) 2. Is there a documented periodic evaluation by external reviewers to validate the internal evaluation process and assess the goal achievement? 3. Are meetings held with stakeholders regularly? 4. Are evaluation results utilized for further corrective actions and planning?		1–4: document analysis

**Table 5:** Checklist for quality practices pertaining to human resources

Human Resources Checklist	Level of Achievement (Fully / Partially / Not Achieved)	Evidence Collection Method
**Faculty**		
*Evaluation questions answered in advance:*		
1. Is there a wide variety of professional development activities for the faculty pertaining to skills needed for online education? 2. Is there a technical checklist for technical support staff? 3. Is there a Standard Operating Procedure (SOP) for technical checks? 4. Are tutor feedback forms available? 5. Are corrective actions taken based on the tutor feedback? 6. Are online teaching activities and experience included in faculty portfolio/appraisal forms? 7. What is the ratio of faculty to students? (one faculty for every 15–20 students) 8. Are the roles and responsibilities of faculty detailed in a document?		1–8: document analysis
*Evaluation questions answered during audit visits:*		
9. Is the faculty roles and responsibilities document available and accessible to faculty? 10. Are faculty aware of the content of their roles and responsibilities document? 11. Are the results of tutor feedback discussed with them? 12. Are the faculty familiar with the different modalities of LMS or online platforms?		9–12: focus groups / observations / interviews
**Students**		
*Information gathered before the audit:*		
1. Is there an orientation session conducted for students regarding the online course? 2. Are learning materials and resources available and accessible during orientation? 3. Are there guidelines for student-teacher and student-student communication means? 4. Are these guidelines disseminated properly?		1–4: document analysis
*Evaluation questions answered during audit visits:*		
5. Are students aware of these guidelines? 6. Are equity and accessibility to technology ensured to all students? 7. Is there a Technical Support office/team available for all students? 8. Does the technical support office offer timely support? 9. Is there timely and constructive feedback to students? 10. Are these guidelines reasonable? 11. Is there a student support unit with a mentorship program? (one staff for each 15–20 student to mentor and guide them)		5, 7, 8, 10: focus groups 6, 9, 11: observations
**Administration**		
*Information gathered before the audit:*		
1. Is there a proper ration between administrative staff and students (Administrators are almost 1 for every 30 students)? 2. Is there an administrator’s training plan? 3. Is there a clear role definition for administration? 4. Is there evidence that the complaint process is used?		1–4: document analysis
*Evaluation questions answered during the audit:*		
5. Are admins trained? Do they possess the required skills? 6. Are admins aware of online course requirements and timelines? 7. Are admins taking the designated training sessions? 8. Do training certificates contribute to admin promotion? 9. Are admin roles disseminated? 10. Are admins aware of these roles? 11. Is there a complaint box / complaint email system? 12. Is the administration supporting the whole process, the faculty members and the students? 13. Is the administration ensuring the availability of resources needed for online teaching different activities? 14. Is the administration monitoring the implementation of online activities with frequent evaluation and actions done for any challenges encountered?		5, 9, 11, 14: observations 6–8, 10, 12, 13: focus groups

The guiding checklist offered in Table 4 is a guide for universities that can be used for self-assessment when intending to evaluate the online educational practices. This checklist can also be adopted by regulating bodies to highlighted required evidence for best practice in online learning.

## Discussion

The use of multi-level analysis to achieve a consensus report is not new to the scientific community and was used by Gorard, 2003; Pahor and Novak, 2017; Shively and Smith, 2019 [11–13]. The main focus of this work was to establish a regional consensus statement around what constitutes effective online teaching practice and thus develop guides to be used when evaluating an online teaching experience. The qualitative nature of the work cherishes the experience of individuals who have utilized crisis management mode post COVID-19 to achieve the most possible educational effectiveness. This experience came with trials and errors and thus lessons that need to be documented and acknowledged. The reliance on expert consensus was used before by Minas and Jorm [14] and Kern [15].

There are three areas of particular concern in developing standards representing best practice in online learning. These were organizational capacity, effective learning and assessment, and human resources. This is in agreement with the developed standards by Kennedy [16], Dawson et al. [17], and Skiba [18].

This categorization laid significant importance on the human resource factor and identified it as a separate standard area in itself. This could be due to the large transition performed by faculty and administration at an unexpected pace after COVID-19. With this transition a lot of human resource adaptation and development was required [19, 20].

Much emphasis was placed on the use and the development of the learning management systems and developing tools to ensure its reliability and user friendliness. This is in agreement with Radwan et al. [21] and Rahrouh et al. [22]. The learning management systems are not intended to be used as repositories for information but rather as a tool to facilitate communication and student engagement [23–26].

This work focuses on the development of material for online teaching and on the difference in adaptations needed to ensure proper learning among students. This agrees with the findings of Bennet and Lockyer [27], Cook and Dupras [28], Kristanto [29], Joseph et al. [27] Mishra et al. [30], Moorhouse [31], and Xhelili et al. [32].

When experts handled the standards, it was obvious that there was an inherent need for quantification in order to set benchmarks. This is supported by the work of many other researchers [24, 25, 27–37]. This fact alone is important to highlight the need for regional standards that adapt easily to the needs of schools in different areas. These standards are thus intended to guide country adaptations and understanding of standards to suit their own practice line.

## Conclusion

Ensuring the quality of online learning is of utmost importance especially during the times of crises [38–40] and dependence totally or partially on online learning. Effort was exerted into experience to apply scientific methodology in identifying the different aspects of best practice descriptors and their success indicators. This included all elements related to online learning environments and processes and all stakeholders involved.

This work provides educators, institutions, and evaluators of educational practices with comprehensive recommendations that address three important axes, which are: a) institutional capacity, b) effective learning and assessment, and c) human resources. Taking such axes into consideration by educators and institutions will lead to planning and implementing successful online learning activities, while taking them into consideration by the evaluators will help them conduct comprehensive audits and provide stakeholders with highly informative evaluation reports.

### Limitations of the study

This work is done in a regional perspective and offers guidance and consensus from a specific region although the findings are generalizable and useful for all regions. The work can be expended and replicated to other regions and for this purpose the authors have made it a mission to describe in great detail the methodology of the work. The sample adopted for this work was a convenience sample which carries the limitation of all similar samples in the sense that there wasn’t a considerable degree of randomization.

Future work can be done to reflect on applicability of the attached checklist in guiding the self-assessment process.

## Supplementary Information

**Additional file 1.**

## Data Availability

The datasets used and/or analyzed during the current study are available at: https://dataverse.harvard.edu/dataverse/online_learning_guide

## Abbreviations

CME: Continuing medical education
ILO: Intended learning outcome
MCQs: Multiple-choice questions
OSCE: Objective Structured clinical examination
OSPE: Objective structured practical examination
DOPs: Direct observation of practical skills
SCU: Supreme Council of Universities
TBL: Team-based learning
