# Supplementary material for: A guide for evaluation of online learning in medical education: a qualitative reflective analysis
Source: BMC Med Educ. 2021 Jun 10;21:339. doi: 10.1186/s12909-021-02752-2 (PMC8190722; doi:10.1186/s12909-021-02752-2)
Supplement: Supplementary file 1 — Additional file 1. [file 12909_2021_2752_MOESM1_ESM.pdf]

Quality Standards for Online Learning in Medical Education

The purpose of this work is to guide the educational shift in medical education through the development of quality standards and indicators. These standards for online education, as a comprehensive set of criteria, are important to establish confidence in online learning among stakeholders and to facilitate structured and objective comparisons between various choices.
The work was approved by the Research and Ethics Committee (REC) of Ain Shams University.
All your data will be used by the researcher of this work.
If you agree to share in this research, please complete this survey
* Required

1. Name

____

2. Affiliation *

____

3. Years of Expertise in Online Education *

____

Online learning quality areas

Which of theses areas are necessary to be evaluated when conducting online learning?

4. Which of theses areas are necessary to be evaluated when conducting online learning? *

Check all that apply.

- [ ] ORGANIZATIONAL CAPACITY (Governance, Resources and Organizational bylaws)
- [ ] EFFECTIVE LEARNING AND ASSESSMENT (Educational Program, Course Design, Course delivery, Student assessment, Evaluation)
- [ ] Human Resources (Faculty, Students and Administration)

Other: [ ] ____________________

5. (Other) Please suggest any additional quality areas that should be added

____

____

____

____

____

Area I. ORGANIZATIONAL CAPACITY

Please select the necessary indicators for each standard that should be evaluated when conducting online learning

6. A. Governance: School Leadership is accountable and committed to support and lead the institution to delivering a high quality online education. *

Check all that apply.

- [ ] Leadership encourages a collaborative environment to plan, implement and monitor the quality of online learning activities
- [ ] Leadership shares and cements the values, beliefs and the operational expectations for a quality online learning
- [ ] Leadership holds themselves accountable to disclose accurate information about the recruitment process, policy, fees, courses/programs, and reports.
- [ ] Leadership demonstrates proactive understanding and analysing organizational needs to deliver effective online education.
- [ ] Leadership creates a culture of acceptance and encouragement for online learning.
- [ ] Leadership delegates responsibility to multidisciplinary teams and facilitates their work to implement and monitor online learning activities.

7. B. Resources: The resources needed for the online learning are allocated in a fair, reasonable manner that responds to the identified needs *

Check all that apply.

- [ ] Presence of a learning management system (LMS) that ensures user-friendly and secure technology enabled learning environment
- [ ] Presence of accessible Internet services
- [ ] Presence of digital tools that are aligned with the educational needs of learners
- [ ] Presence of the equipment that support successful online learning
- [ ] Presence of trained technical support team
- [ ] Financial resources allocated to online learning
- [ ] Provisional needs documents are available designed annually and approved by proper authorities.
- [ ] Budget is well-managed in a transparent and documented way
- [ ] Option 9

8. C. Organizational bylaws: The bylaws clearly define the administrative issues, credit points calculation and the roles and responsibilities of team members *

Check all that apply.

- [ ] Presence of written policies & procedures or all online courses
- [ ] There is a defined and documented process related to the online programs
- [ ] There is a documented clear policy governing the ongoing training and support to the working staff
- [ ] All students have equitable access to the online learning resources

9. (Brainstorming) Please list any more standards or indicators you think can be added under the organizational capacity area.

10. If you have any additional comments on the organizational capacity area, please include them below

Area II. EFFECTIVE LEARNING AND ASSESSMENT

Please select the necessary indicators for each standard that should be evaluated when conducting online learning

11. D. Educational Program: The program has a clear robust design that respects the school vision, mission and values and that demonstrates a clear understanding of the nature of the required graduate attributes. *

Check all that apply.

- [ ] There is an approved, updated and well-constructed longitudinal online education plan that includes sufficient data to support decisions and is aligned with the educational program
- [ ] There are aligned and cascaded goals: strategic, long term, intermediate and short-term goals
- [ ] There is clear identification of the required resources to ensure sustainability of the online programs and courses

12. E. Course Design: The courses have a clear robust design that respects the school vision, mission and values with a clear distinction of the allocation of online teaching/ learning practices. *

Check all that apply.

- [ ] Courses have clearly stated learning objectives/ competencies that are aligned with the organization goals
- [ ] The course learning objectives or competencies describe outcomes that are measurable
- [ ] The selected contents are UpToDate, related to learning goals and follow the legal requirements (ownership, intellectual property, copyrights)
- [ ] The instructional materials contribute to the achievement of the stated learning objectives or competencies
- [ ] Online instructional methods and tools support active learning , student involvement, support interaction amongst students and between instructors and students and are based on recent best practices
- [ ] Online instructional methods are variable and support development of higher order thinking
- [ ] The relationship between learning objectives or competencies and course activities is clearly stated
- [ ] There is use of digital tools that best support students' involvement and better understanding of the learning material
- [ ] Learning and assessment schedules are clear, applicable and fair for all students
- [ ] Online student Assessment methods planned are clear, fair for all students and include frequent formative assessment with feedback and summative assessment with clear and transparent reporting

13. F. Course delivery: Courses should be delivered in the safest most accessible way providing standardised learning opportunities *

Check all that apply.

- [ ] There is a plan for frequent evaluation that is approved and implemented with identified data collection methods eg. observation, questionnaires, focus group
- [ ] There is a well organized plan for delivery with a back up
- [ ] A troubleshooting and complaint policy and procedure exists and is announced and used by learners
- [ ] Designed learning activities are implemented with minimal deviation from plans
- [ ] Technologies required in the course are readily obtainable.
- [ ] Option 6

14. G. Student assessment: The student assessment to measure student achievement using multiple methods that align with the learning objectives and the instructional methods. Data from assessment is evaluated and feeds into educational decision making. *

Check all that apply.

- [ ] Digital tools are used to ensure secure, fair, valid and applicable assessment
- [ ] There is use of multiple assessment methods to measure cognition, skills and attitude of the students
- [ ] There is use of frequent formative assessment with feedback for better learning
- [ ] There are clear reports after the summative assessment
- [ ] There is a plan for academic counselling that is clear, manageable and is executed.

15. H. Evaluation: The educational monitoring and evaluation plans are available with clearly assigned evaluation questions, key performance indicators and assigned personnel. The plan is implemented and the information it generates feeds into the educational re-planning

Check all that apply.

- [ ] There is documented continuous monitoring and evaluation for the online learning materials/ process by internal reviewers to collect and analyze data for continuous improvement. (about LMS, Faculty performance and satisfaction, and Students' Engagement, Satisfaction, and Achievement)
- [ ] There is documented periodic evaluation by external reviewers to validate the internal evaluation process and assess the goal achievement
- [ ] There is disclosure of the evaluation results with the stakeholders
- [ ] Data is used to drive decisions for continuous improvement

16. (Brainstorming) Please list any more standards or indicators you think can be added under the Effective Learning and Assessment Area

17. If you have any additional comments on the Effective Learning and Assessment Area, please include them below

Area III. Human Resources: The organization has personnel who can manage the educational process effectively and who are under continuous monitoring and development.

Please select the necessary indicators for each standard that should be evaluated when conducting online learning

18. 1- Faculty / Staff *

Check all that apply.

- [ ] There is a wide variety of professional development activities for the faculty pertaining to skills needed for online education
- [ ] There is timely and effective technical support to the faculty
- [ ] There is timely, frequent and constructive feedback about instructor performance
- [ ] Faculty have an opportunity to add to their professional portfolio within online learning in the school
- [ ] The number of assigned faculty is reasonable, sufficient and aligned with the student number and educational activities
- [ ] There is a clear definition of faculty roles and responsibilities

19. Students *

Check all that apply.

- [ ] There is briefing and orienting the students about the accessibility and availability of the online learning resources and digital tools
- [ ] Equity and accessibility to technology to all students is ensured
- [ ] There is timely and effective technical support to students to overcome limitations of technology & computer literacy
- [ ] There is timely and constructive feedback to students
- [ ] There are guidelines for student-teacher and student-student communication

20. Administration *

Check all that apply.

- [ ] There exists a supporting administration team that is reasonable and aligned with the educational processes, number of students, number of faculty etc.
- [ ] There is a solid development plan for administration of the online learning program
- [ ] There is a clear role definition for administration
- [ ] There is a definite pathway for troubleshooting and for complaints for administrators in the program

21. (Brainstorming) Please list any more standards or indicators you think can be added under the Resources Area

22. If you have any additional comments on the Resource Area, please include them below

This content is neither created nor endorsed by Google.

Google Forms
